# The Role of Zinc in Pediatric Respiratory Infections: Evidence from Clinical Trials and Real-World Studies

**DOI:** 10.3390/nu18040557

**Published:** 2026-02-07

**Authors:** Giulio Dinardo, Cristiana Indolfi, Angela Klain, Carolina Grella, Maria Angela Tosca, Michele Miraglia del Giudice, Giorgio Ciprandi

**Affiliations:** 1 Department of Woman, Child and General and Specialized Surgery, University of Campania “Luigi Vanvitelli”, 80138 Naples, Italy; dinardogiulio@gmail.com (G.D.); klainangela95@gmail.com (A.K.); caro.grella94@gmail.com (C.G.); michele.miragliadelgiudice@unicampania.it (M.M.d.G.); 2 Allergy Center, IRCCS Istituto Giannina Gaslini, 16147 Genoa, Italy; mariangelatosca@gaslini.org; 3 Allergy Clinic, Casa di Cura Villa Montallegro, 16145 Genoa, Italy; gio.cip@libero.it

**Keywords:** zinc, pediatric infections, respiratory tract infections, recurrent respiratory infections, tonsillitis, pneumonia, bronchiolitis, immunonutrition, dietary supplements, children

## Abstract

**Background/Objectives:** Zinc is an essential trace element involved in multiple aspects of immune function, including epithelial barrier integrity, innate and adaptive immune responses, regulation of inflammation and oxidative stress. Zinc deficiency has been associated with increased susceptibility to infections, particularly in the pediatric population. This narrative review aims to summarize and discuss current evidence on the role of zinc in the prevention and management of pediatric respiratory infections. **Methods:** A comprehensive literature review was conducted including randomized controlled trials, real-world studies, and international guidelines published in recent years. Both zinc monotherapy and multicomponent dietary supplements containing zinc were considered. **Results:** Evidence consistently supports a preventive role of zinc supplementation in reducing the incidence and burden of respiratory infections, particularly in children with recurrent disease and in zinc-deficient populations. Zinc-containing multicomponent supplements demonstrated significant reductions in infection frequency and duration, alongside improved patient and parent-reported outcomes, with a favorable safety profile. In contrast, data on zinc as an adjunctive treatment during acute infections, especially severe pneumonia, are less consistent, with limited impact on major clinical outcomes. The effectiveness of zinc appears to be influenced by treatment duration, baseline nutritional status, and formulation. **Conclusions:** In conclusion, zinc may represent a valuable component of preventive immune-nutritional strategies for pediatric respiratory infections, especially when administered as part of multicomponent formulations and over prolonged periods. While its role in acute disease management remains uncertain, optimizing zinc status may contribute to reducing infection recurrence and overall disease burden. Further well-designed trials are warranted to clarify optimal dosing, timing, and target populations.

## 1. Introduction

Respiratory tract infections (RTIs) represent one of the leading causes of morbidity and healthcare utilization in childhood, ranging from self-limiting upper respiratory tract infections to potentially life-threatening conditions such as pneumonia. The global burden of RTIs remains especially relevant in low- and middle-income settings but continues to be substantial across all pediatric populations, contributing to significant antibiotic exposure, recurrent medical visits, school absenteeism, and impaired quality of life [1,2]. Over the past decade, growing interest has focused on the role of nutritional factors in modulating susceptibility to respiratory infections, particularly micronutrients involved in immune homeostasis. Among these, zinc has emerged as a biologically plausible and clinically promising candidate [3,4].

### 1.1. Mechanisms of Action of Zinc in Infectious Respiratory Diseases

Zinc influences respiratory infections through multiple, interrelated mechanisms that span barrier protection, innate and adaptive immunity, antiviral and antibacterial pathways, and control of inflammation. At the epithelial level, zinc maintains tight-junction integrity and supports mucociliary clearance, thereby limiting pathogen adherence and invasion. Deficiency disrupts epithelial repair and increases permeability, favoring colonization [5,6].

**Figure 1:**
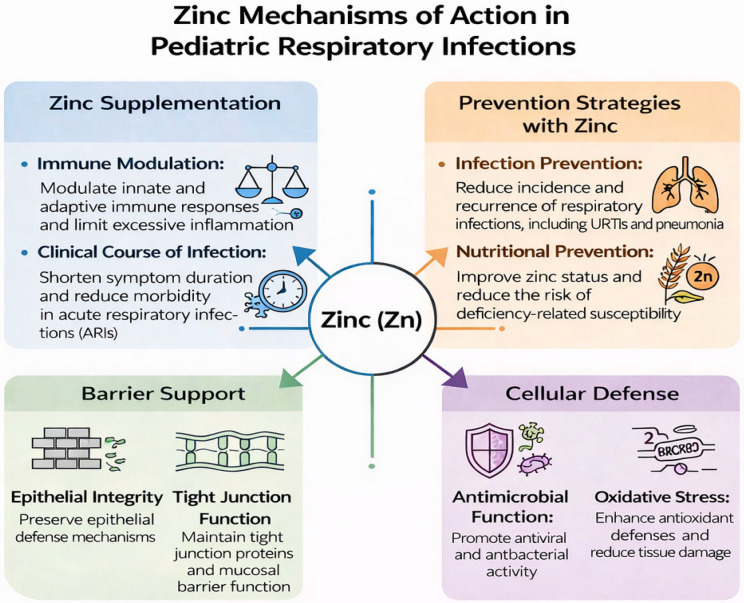
Mechanisms of action of zinc in pediatric respiratory infections.

Within innate immunity, zinc modulates neutrophil chemotaxis, macrophage phagocytosis, and natural killer cell cytotoxicity, while participating in the regulation of pattern-recognition receptor signaling. Zinc acts as a “second messenger” in immune cells, shaping downstream transcriptional programs essential for antimicrobial defense [7]. It also promotes production of antimicrobial peptides such as cathelicidins and defensins, enhancing early containment of viral and bacterial replication [6,7]. In adaptive immunity, zinc is critical for thymic function, T-cell differentiation, and balanced Th1/Th2 responses. Insufficient zinc impairs T-cell activation, reduces interleukin-2 synthesis, and compromises antibody responses, contributing to recurrent and more severe infections. Conversely, adequate zinc availability supports effective yet controlled immune activation. Zinc also exhibits direct antiviral and antibacterial effects. In vitro data suggest that zinc interferes with viral RNA polymerase activity, inhibits viral entry and uncoating, and limits replication of several respiratory viruses, including rhinovirus and coronaviruses [6]. Moreover, zinc may attenuate bacterial virulence and biofilm formation. Importantly, these antimicrobial effects operate alongside immunomodulatory properties that restrain excessive inflammation, a key driver of tissue damage in lower respiratory infections. Finally, zinc participates in oxidative stress balance by stabilizing cellular membranes and acting as a cofactor for antioxidant enzymes such as superoxide dismutase. Through this combined antioxidant and anti-inflammatory action, zinc may reduce cytokine overproduction and accelerate clinical recovery, as suggested in pediatric pneumonia and recurrent infection trials [5,8,9] (Figure 1). Together, these mechanisms provide a coherent biological rationale linking zinc status with susceptibility, severity, and outcomes of pediatric respiratory infections, and they underpin the clinical findings discussed in the subsequent sections of this review.

### 1.2. Clinical Relevance of Zinc in Childhood Respiratory Infections

Zinc is an essential micronutrient involved in more than 300 enzymatic reactions and numerous transcriptional processes. In early life, adequate zinc status is crucial for normal growth, thymic development, and maturation of both innate and adaptive immune responses [10]. In pediatric age, inadequate zinc intake or marginal deficiency is common, especially in conditions of malnutrition, dietary restriction, chronic disease, or infection-related loss [11]. It is important to note that the pediatric population spans a wide age range (0–18 years), encompassing neonates, infants, toddlers, children, and adolescents, whose immune responses and zinc requirements differ substantially. Neonates and infants have immature immune systems and may be particularly vulnerable to zinc deficiency, whereas older children and adolescents have distinct nutritional needs and more mature immune function. Consequently, the effects of zinc supplementation may vary by age group, and interpretation of studies pooling broad pediatric cohorts should be done with caution [12].

Observational studies consistently demonstrate that children with lower zinc concentrations exhibit higher susceptibility and more severe clinical courses of RTIs, including lower respiratory tract infections [11,13]. Randomized controlled trials and meta-analyses have increasingly explored the preventive and therapeutic role of zinc. Preventive supplementation programs have shown reductions in infectious morbidity, particularly in regions with endemic deficiency [12,14]. Similarly, zinc-fortified foods and biofortification programs have been proposed as population-level interventions capable of improving zinc status and, indirectly, infection outcomes [15,16]. At the same time, controlled trials evaluating zinc as an adjunct treatment for acute respiratory infections, have reported faster clinical recovery in selected pediatric cohorts, though heterogeneity across studies remains substantial [8,17,18].

Upper airway diseases provide additional insight into the potential clinical value of zinc. Studies on recurrent respiratory infections and tonsillopharyngitis suggest that multicomponent supplements containing zinc, often in combination with vitamin D, β-glucans, *Pelargonium sidoides*, propolis, and honey, may reduce symptom burden, recurrence, and need for rescue medication, particularly when used as adjuvant therapy rather than as stand-alone treatment [9,19,20]. These findings align with broader evidence supporting zinc’s capacity to modulate innate antiviral responses, enhance mucosal immunity, and attenuate inflammatory injury [6,7]. Importantly, systematic reviews and Cochrane analyses, reinforce that zinc supplementation may reduce the incidence and duration of respiratory infections in children, especially where baseline deficiency is likely, while also acknowledging variability related to formulation, dosing, timing, and population characteristics [18,21,22].

It should be acknowledged that zinc is often discussed in clinical studies and reviews as a single entity, despite the existence of different zinc salts (e.g., zinc sulfate, gluconate, acetate) and delivery vehicles that may influence bioavailability, gastrointestinal tolerability, and systemic absorption. Variability in chemical form, formulation, and administration matrix (e.g., syrups, tablets, fortified foods, or multicomponent supplements) may partly contribute to the heterogeneity observed across clinical trials, particularly with respect to efficacy outcomes. However, most pediatric studies do not provide sufficient detail or comparative analyses to allow stratification of results according to zinc salt or delivery system, limiting the ability to draw formulation-specific conclusions [21].

Synthesizing the available studies, existing evidence supports a dual conceptual framework: zinc as a preventive strategy aimed at restoring optimal immune competence and a therapeutic adjunct capable of accelerating recovery and mitigating complications in selected infections. However, unresolved questions persist. These include the optimal dosing regimen, duration of supplementation, criteria for patient selection, interactions with other micronutrients, and the relative contribution of zinc when included within multicomponent nutraceutical formulations. Furthermore, most available studies are heterogeneous in design and outcomes, and many derive from high-risk populations, limiting generalizability [21,22].

The present narrative review aims to synthesize current mechanistic and clinical evidence regarding the role of zinc in pediatric respiratory infections, integrating findings from experimental models, randomized controlled trials, observational studies, and large meta-analyses. Particular emphasis is placed on the differential contribution of zinc to prevention strategies, such as supplementation and its application as adjunctive therapy in acute infections including bronchiolitis, pneumonia, and tonsillopharyngitis.

## 2. Materials and Methods

A bibliographic search was conducted using PubMed and the Cochrane Library to identify studies published in English between 2015 and 2025 investigating the role of zinc in pediatric respiratory infections. Both observational and interventional studies involving human subjects were considered eligible. The search strategy was developed using a combination of Medical Subject Headings (MeSH) terms and free-text keywords related to zinc, respiratory tract infections, and pediatric populations. The following search string was applied in PubMed: ((“zinc” [MeSH Terms] OR “zinc” [All Fields]) AND (“respiratory tract infections” [MeSH Terms] OR “respiratory tract infections” [All Fields]) AND (“child” [MeSH Terms] OR “adolescent” [MeSH Terms])).

**Figure 2:**
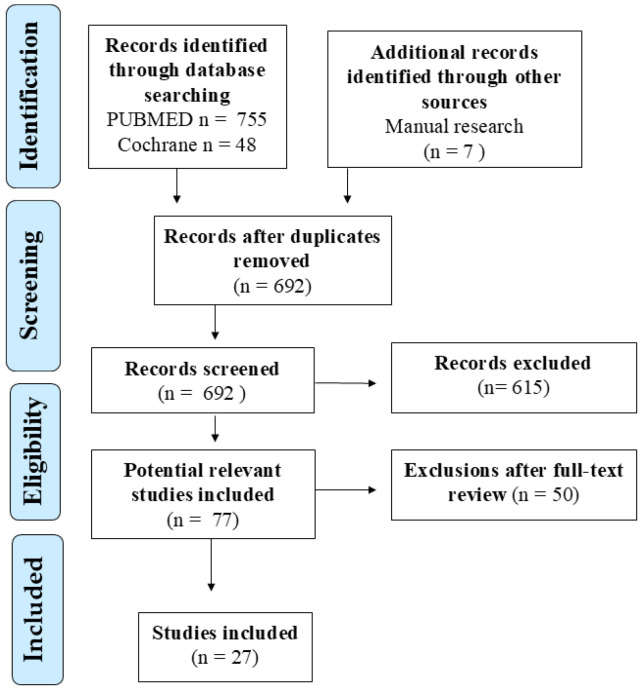
Flow diagram of the literature search and study selection process.

Studies were included if they involved children (0–11 years) or adolescents (12–18 years) and reported data on zinc status, zinc deficiency, or zinc supplementation in relation to respiratory infections. Eligible outcomes included incidence, duration, severity, recurrence, and prevention of upper or lower respiratory tract infections, including common cold, tonsillopharyngitis, bronchiolitis, and pneumonia. Both zinc administered as monotherapy and zinc-containing multicomponent dietary supplements were considered. Studies focusing exclusively on adults, non-respiratory infections, in vitro/animal models, or unrelated clinical outcomes were excluded. After duplicate removal, all retrieved records were screened by title and abstract. Full-text articles were independently reviewed by two authors (G.D. and C.I.). Disagreements were resolved through consensus and, when necessary, consultation with a third reviewer (M.M.d.G.). Studies that were retracted, lacked accessible full text, or failed to meet inclusion criteria were excluded. This work was designed as a narrative review with a structured literature search, aimed at providing a clinically oriented synthesis of current evidence. Although database searching and study selection steps were performed, no formal risk-of-bias assessment or meta-analysis was conducted. However, greater interpretative weight was given to randomized controlled trials, Cochrane reviews, and large meta-analyses, while real-world and observational studies were considered hypothesis-generating (Figure 2).

## 3. Results

### 3.1. Role of Zinc in Acute Bronchiolitis

Zinc is a key micronutrient supporting immune function and epithelial integrity, with potential relevance in acute viral lower respiratory infections such as bronchiolitis. It helps maintain the respiratory barrier, modulates immune responses, and exerts antioxidant and anti-inflammatory effects, while experimental evidence suggests it may limit viral replication and promote mucociliary clearance and epithelial repair [18,23,24]. Clinical evidence on zinc supplementation in acute bronchiolitis, however, remains inconsistent. A double-blind, placebo-controlled trial conducted in infants with acute bronchiolitis reported that oral zinc sulphate administration was associated with a faster resolution of clinical symptoms, including cough and wheezing, as well as a shorter duration of hospitalization compared with placebo. These findings suggested a potential adjunctive benefit of zinc when added to standard supportive care, without significant adverse effects [24]. In contrast, more recent randomized controlled trials have failed to demonstrate a significant improvement in key clinical outcomes, such as respiratory rate, wheezing severity, oxygen saturation, or length of hospital stay, in infants receiving zinc supplementation compared with controls [23]. Several factors may explain these divergent results, including differences in study design, zinc dosage, timing of administration, baseline zinc status, and disease severity. Bronchiolitis is typically an acute, self-limited viral illness, and the rapid progression of symptoms may limit the window during which zinc-mediated immunomodulatory effects can translate into measurable clinical benefits [23,24]. Moreover, during acute infection, zinc redistribution from the circulation to the liver may reduce its bioavailability at the site of infection, potentially attenuating its therapeutic impact [23]. While a few trials reported modest improvements in symptom resolution or duration of hospitalization, the majority of randomized controlled studies failed to demonstrate significant benefits in key clinical outcomes. Factors such as differences in study design, zinc dosage, timing of administration, baseline zinc status, and the self-limiting nature of the illness may contribute to these discrepancies. Overall, current evidence does not support routine zinc supplementation as a therapeutic strategy in acute bronchiolitis [18,23,24].

### 3.2. Zinc and Tonsillitis

Zinc has been increasingly investigated in the context of recurrent tonsillitis due to its central role in immune regulation and mucosal defense within the Waldeyer’s ring. Evidence from tissue-based studies indicates that children with recurrent tonsillitis exhibit significantly lower zinc concentrations in palatine tonsillar tissue compared with those with tonsillar hypertrophy, suggesting that local zinc deficiency may impair immune responses and increase susceptibility to repeated infections [25]. These findings support the hypothesis that adequate zinc availability is crucial for maintaining tonsillar immune competence and controlling inflammatory processes. Clinical data further suggest a potential therapeutic role for zinc-containing supplements in recurrent tonsillitis [25]. A randomized controlled trial evaluating an oral immunomodulatory supplement containing zinc, along with vitamins and other immune-supportive compounds, demonstrated a significant reduction in the frequency and severity of tonsillitis episodes, fever recurrence, and tonsillar volume in treated children compared with controls [19]. Although the observed benefits cannot be attributed exclusively to zinc due to the multi-component formulation, the known effects of zinc on macrophage function, antigen presentation, and cytokine signaling provide a plausible biological contribution to the reported clinical improvements [19]. Overall, current evidence suggests that zinc deficiency may represent a predisposing factor for recurrent tonsillitis, while zinc supplementation, particularly as part of combined immunonutritional strategies, may help reduce disease recurrence.

### 3.3. Zinc and Pediatric Pneumonia

Zinc deficiency is widely recognized as a significant risk factor for pediatric pneumonia, particularly in low- and middle-income countries where inadequate dietary intake and recurrent infections are common [26]. Zinc plays a crucial role in both innate and adaptive immune responses by supporting epithelial barrier integrity, neutrophil and macrophage function, cytokine signaling, and lymphocyte proliferation. These biological functions provide a strong rationale for investigating zinc supplementation as a strategy to reduce pneumonia-related morbidity and mortality in children. Epidemiological and interventional studies have consistently shown that long-term zinc supplementation is associated with a reduced incidence of pneumonia and acute lower respiratory tract infections in children aged 2 to 59 months, especially when administered for periods longer than three months in zinc-deficient populations [8,17,18].

In contrast, the therapeutic role of zinc during acute pneumonia episodes remains controversial. Multiple randomized controlled trials and meta-analyses consistently demonstrate that adjunctive zinc supplementation does not significantly improve clinical outcomes in children with acute pneumonia, including mortality, treatment failure, length of hospitalization, or resolution of major clinical signs. While some studies reported modest improvements in secondary outcomes, these effects were inconsistent and do not support routine use of zinc as adjunctive therapy [17]. Overall, available evidence suggests that adjunctive zinc therapy does not substantially modify the clinical course of severe pediatric pneumonia [8].

The discrepancy between preventive and therapeutic efficacy may be partly explained by the pathophysiology of acute pneumonia. During severe infection, zinc is rapidly redistributed from the circulation to the liver as part of the acute-phase response, potentially limiting its bioavailability at the site of infection. One proposed explanation involves redistribution of zinc to hepatic stores during the acute-phase response; however, this mechanism remains hypothesis-generating rather than directly demonstrated in pediatric pneumonia trials. Moreover, the rapid progression of pneumonia and the dominance of pathogen-specific and inflammatory pathways may reduce the capacity of short-term zinc supplementation to meaningfully influence disease course [8,17]. Consequently, zinc supplementation administered during acute illness appears insufficient to overcome the underlying immune dysregulation associated with established pneumonia [8,17]. Reflecting this evidence, the most recent World Health Organization (WHO) update on childhood pneumonia and diarrhea emphasizes zinc supplementation primarily as a preventive public health intervention rather than a component of acute pneumonia management. WHO recommendations highlight the importance of improving nutritional status, including adequate zinc intake, to reduce the overall burden of pneumonia, while not endorsing routine adjunctive zinc therapy during acute pneumonia episodes in otherwise adequately treated children [26]. Taken together, current data support a clear distinction between the preventive and therapeutic roles of zinc in pediatric pneumonia, underscoring its value in reducing disease incidence and severity at the population level, while providing limited justification for its routine use as an adjunctive treatment during established disease.

### 3.4. Zinc-Containing Multicomponent Supplements in Pediatric Upper Respiratory Infections

Although zinc has emerged as a promising nutraceutical in pediatric respiratory health, many commercially available products combine zinc with multiple bioactive compounds. In line with this trend, several clinical studies have investigated multicomponent formulations that include zinc as part of broader preventive or supportive strategies for Upper Respiratory Infections (URTIs) in children [27,28,29]. In a real-world primary care study, supplementation with Stimunex^®^, a formulation including zinc, β-glucan, vitamin D3, and Sambucus nigra extract, led to a significant reduction in both the number and duration of respiratory infections compared with standard care alone, with benefits extending to both upper and lower respiratory tract episodes and without relevant adverse effects [9]. Notably, the greatest effects were observed during the follow-up period, suggesting that prolonged supplementation may gradually enhance immune competence through synergistic modulation of innate and adaptive immune responses. Comparable findings have emerged in randomized controlled trials addressing recurrent tonsillitis. In the Difensil Immuno study, a zinc-containing immunonutritional supplement significantly decreased recurrence rates, symptom burden, and tonsillar inflammation, supporting the hypothesis that zinc contributes to improved mucosal immunity and epithelial barrier stability while attenuating excessive inflammatory responses of the upper airways [19]. These observations are biologically plausible, given the central role of zinc in immune cell maturation, cytokine signaling, and antimicrobial defense mechanisms. Additional evidence derives from the PediaFlù randomized controlled trial protocol, designed to evaluate zinc-containing supplementation as an adjunct to standard therapy in acute tonsillopharyngitis and rhinopharyngitis. The study emphasizes patient-centered outcomes such as symptom duration, clinical recovery, and quality-of-life parameters, highlighting the growing interest in complementary therapeutic strategies during acute infectious events [20]. However, despite promising signals, important limitations must be acknowledged. Because most available data concern multicomponent formulations, it remains difficult to isolate the specific contribution of zinc from other active constituents such as β-glucans, vitamin D, or plant-derived polyphenols. Moreover, real-world, open-label designs and parental reporting of outcomes introduce potential biases and limit causal inference.

Overall, the current body of evidence suggests that zinc-containing multicomponent supplements may be particularly valuable in children with recurrent respiratory infections, where preventive immunomodulation appears more effective than short-term adjunctive therapy in acute illness. It is important to note that many positive outcomes in these studies cannot be attributed solely to zinc, as the supplements also contain vitamins, β-glucans, and plant extracts. Therefore, caution is needed when interpreting efficacy data from multicomponent interventions, and findings from zinc monotherapy trials should be considered separately. Nevertheless, further rigorously designed randomized controlled trials are required to clarify optimal dosing, duration, formulation characteristics, and target populations, and to better disentangle the specific role played by zinc within these complex nutritional preparations.

## 4. Discussion

The present narrative review highlights the complex and multifaceted role of zinc in pediatric respiratory infections, encompassing prevention, adjunctive treatment, and broader immune nutritional strategies. Across the included literature, zinc emerged as a micronutrient with clear biological plausibility and variable clinical performance, largely influenced by population characteristics, baseline nutritional status, clinical setting, and formulation.

From a preventive perspective, zinc supplementation has consistently demonstrated meaningful reductions in the incidence and recurrence of respiratory tract infections in children, particularly in populations at nutritional risk or with recurrent respiratory disease phenotypes. Zinc exerts multiple interrelated biological effects that support immune defense and epithelial integrity, including modulation of innate and adaptive immunity, maintenance of mucosal barrier function, antiviral and antibacterial activity, and antioxidant and anti-inflammatory properties. These mechanisms provide a coherent rationale for its preventive role in pediatric respiratory infections and underpin the clinical outcomes observed across trials. Evidence from controlled and real-world settings suggests that sustained supplementation strengthens mucosal defense mechanisms, modulates immune responses, and reduces infection burden over time [12,18,30]. These findings are echoed in primary care data where multicomponent products containing zinc, vitamin D, β-glucans, and plant extracts reduce both frequency and duration of recurrent infections [9]. Taken together, these observations reinforce the concept that zinc is best conceptualized as an immunonutrient whose benefits are cumulative and preventive rather than acutely therapeutic.

### 4.1. Differential Roles of Zinc: Preventive Versus Therapeutic Effects

A consistent finding across the available literature is the need to distinguish the preventive effects of zinc from its potential therapeutic role during acute respiratory infections. Preventive supplementation, particularly when administered over extended periods and in settings characterized by suboptimal nutritional status, is associated with a reduction in infection susceptibility, recurrence, and overall morbidity. This pattern is evident in studies evaluating zinc as part of nutritional or public-health interventions, where supplementation has been linked to decreased frequency and duration of both upper and lower respiratory tract infections, especially in populations at higher risk of deficiency (Table 1).

In contrast, the evidence regarding the therapeutic administration of zinc during established infections remains heterogeneous. Randomized controlled trials examining adjunctive zinc therapy in children with severe pneumonia generally failed to demonstrate clinically meaningful reductions in mortality, treatment failure, or length of hospitalization [8,17]. Although modest improvements in select secondary outcomes have occasionally been observed, these findings have not translated into consistent therapeutic benefit. From a mechanistic perspective, the acute-phase response associated with infection induces a redistribution of zinc from the circulation to hepatic stores, thereby reducing its short-term bioavailability and potentially attenuating the immediate effect of supplementation during acute illness (Table 2).

Notwithstanding these limitations, some interventional data suggest context-dependent benefit. Preventive supplementation in community settings has been associated with lower rates of respiratory and gastrointestinal infections in children [14], and a randomized trial in Thai children reported shorter symptom duration in specific subgroups with acute respiratory tract infection [31]. Furthermore, adult studies have suggested a reduction in common cold duration with zinc administration [32], although extrapolation to pediatric populations requires caution. Recurrent and chronic-relapsing phenotypes appear particularly responsive to zinc-based strategies. Reduced tissue zinc concentrations have been documented in children with recurrent tonsillar disease, supporting a potential link between local micronutrient homeostasis and mucosal immune competence. Clinical trials investigating multicomponent nutraceutical formulations containing zinc have demonstrated reductions in recurrence rates, symptom burden, and healthcare resource utilization in children with recurrent respiratory infections and tonsillitis [9,19]. These observations suggest that zinc may exert beneficial effects by supporting epithelial barrier integrity, modulating inflammatory pathways, and enhancing innate antimicrobial responses when used as part of broader preventive regimens. Overall, the current body of evidence supports a differentiated interpretation of zinc supplementation. Its most consistent role appears to lie in prevention and in the long-term modulation of susceptibility to respiratory infections, particularly in children at risk of deficiency or recurrent disease. By contrast, its function as an adjunctive treatment during acute episodes remains uncertain and likely dependent on clinical context, baseline zinc status, and timing of administration. Further rigorously designed studies are required to clarify these aspects and to better define patient selection, optimal dosing, and clinically relevant outcomes.

**Table 1:** Zinc for Prevention and Reduction in Recurrence in Pediatric Respiratory Infections.

First Author (Year)	Study Type	Population	Clinical Condition	Zinc Intervention (Formulation Dose-Duration)	Baseline Zinc Status Assessed	Key Findings & Conclusion
Khera (2020) [33]	Interventional Study	Children 6 mo–5 yrs	Acute Respiratory Infection (ARI) prevention	Elemental zinc 20 mg/day—14 days (in deficient children only)	Yes	Marked reduction in Acute Upper Respiratory Infection (AURI) and Acute Lower Respiratory Infection (ALRI) incidence and duration. Benefit strongest in deficient children.
Martinez-Estevez (2016) [14]	Randomized controlled trial	School-age children	Prevention of Respiratory Tract Infection (RTI) & diarrhea	Elemental zinc 10 mg/day—several months	No	Fewer infectious episodes and improved morbidity indicators.
Lassi (2016) [18]	Randomized controlled trial	Children 2–59 mo	Prevention of pneumonia	Typically 10 mg/day (6–11 mo) and 20 mg/day (>12 mo)—4–6 months	Not reported	Reduced pneumonia incidence, especially in high-risk settings.
Imdad 2023 [12]	Systematic review	Children 6 mo–12 yrs	Mortality, morbidity, RTI	Varied across trials (5–20 mg/day, weeks–months)	Yes	Preventive zinc reduces infection burden most where deficiency likely.
Kujinga 2018 [15]	Randomized trial	Preschool children	Prevention	Zinc-fortified water	Yes	Improved zinc status and reduced infection-related morbidity.
Lowe 2020 [16]	Interventional Study	Population-based, primary care/school-based setting	Nutritional deficiency/infection risk	Dietary zinc enhancement through food biofortification/fortification	Yes	Feasible public-health strategy potentially lowering RTI burden.
Giannattasio 2022 [9]	Real-world prospective	Children with recurrent RI	Recurrent Upper and Lower Respiratory Tract Infections (URTI/LRTI)	Multicomponent (includes zinc)—1 drop/kg/day for 1 month, then 15 days/month × 3 months	Not reported	Fewer infections, shorter duration, excellent tolerability.
Stadio 2020 [19]	Randomized controlled trial	Children	Recurrent tonsillitis	Multicomponent incl. zinc—weeks–months	Not reported	Reduced recurrence and severity; likely mucosal immune support.

Abbreviations: ARI, acute respiratory infection; AURI, acute upper respiratory infection; ALRI, acute lower respiratory infection; RTI, respiratory tract infection; URTI, upper respiratory tract infection; LRTI, lower respiratory tract infection; RI, respiratory infection; RCT, randomized controlled trial; SoC, standard of care; mo, months; yrs, years.

**Table 2:** Zinc as an Adjunct to Standard Therapy in Acute Pediatric Respiratory Infections.

First Author (Year)	Study Type	Population	Clinical Condition	Zinc Intervention (Formulation Dose-Duration)	Baseline Zinc Status Assessed	Key Findings & Conclusion
Wang 2018 [8]	Randomized controlled trial	Hospitalized children	Severe pneumonia	Elemental zinc (commonly 20 mg/day)—during hospitalization	**No**	No benefit on mortality, treatment failure, or hospital stay.
Sakulchit 2017 [17]	Randomized controlled trial	Children with pneumonia	Community-acquired pneumonia	Adjunct zinc in syrup form 10–20 mg/day	**Not reported**	Mixed or neutral outcomes despite biochemical improvements.
Abolfazl Mahyar (2016) [24]	Double-blind RCT	Infants	Bronchiolitis	Elemental zinc liquid—10 mg/day	**No**	No consistent reduction in time to recovery.
Rerksuppaphol 2019 [31]	Randomized controlled trial	Hospitalized children 2–60 mo	Acute lower respiratory infection (ALRI)	Elemental zinc syrup 30 mg/die—7–14 days	**Not reported**	Faster resolution of ALRI and shorter hospital stay vs. placebo
Cardinale 2024 [20]	Randomized (protocol)	Children	Acute tonsillopharyngitis/rhinopharyngitis	Multicomponent oral solution (Pelargonium, propolis, honey, zinc 13 mg/100 mL)—6 days	**No**	Designed as add-on to SoC; outcomes pending.

### 4.2. Multicomponent Formulations and Real-World Relevance

A considerable proportion of the current evidence base derives from studies evaluating multicomponent dietary supplements rather than zinc monotherapy. These preparations typically combine zinc with other bioactive substances, including vitamin D, β-glucans, plant extracts, and additional micronutrients, and appear particularly promising in children with recurrent respiratory infections [4]. Their effects seem to extend beyond direct antimicrobial activity, supporting mechanisms such as enhancement of innate immune responses, stabilization of epithelial barrier function, and modulation of inflammatory pathways, as suggested by real-world observations in primary care settings [3,9,34]. Similar preventive signals emerge from nutritional approaches aimed at improving zinc intake through fortified vehicles, such as drinking water, which have demonstrated improvements in zinc status and reductions in infection-related morbidity in pediatric populations [15].

The relevance of these findings is further reflected in protocol-driven clinical research evaluating zinc-containing supplements as adjuncts during acute upper respiratory conditions, including tonsillopharyngitis [20]. Although definitive results are still evolving, such studies illustrate a growing interest in integrative strategies aimed at improving recovery trajectories while minimizing unnecessary pharmacologic exposure. From a broader public-health perspective, population-level strategies also deserve attention. Agricultural interventions such as biofortification programs have demonstrated that increasing dietary zinc content at scale may contribute to improved nutritional status and potentially reduce infection burden among children [16]. These approaches highlight the complementary role of structural nutritional policies alongside clinical supplementation. An additional critical aspect concerns the possibility of synergistic or antagonistic interactions among components of multicomponent formulations. Zinc may act synergistically with vitamin D in supporting immune modulation and epithelial barrier integrity, while β-glucans may enhance innate immune priming. Conversely, overlapping immunomodulatory pathways or competitive intestinal absorption among micronutrients could attenuate zinc-specific effects. The lack of factorial or component-resolved clinical trial designs limits the ability to determine whether observed benefits are attributable to zinc itself or to the combined biological activity of the formulation. Despite these encouraging signals, interpretation must remain cautious. The specific contribution of zinc is often difficult to isolate within multicomponent formulations, and real-world studies are inherently vulnerable to confounding, reporting bias, and variability in diagnostic definitions. Nevertheless, these data provide valuable ecological insight, complement randomized controlled trials, and reinforce the concept that optimizing zinc intake, whether through supplementation, fortified foods, or broader nutritional policies may represent a relevant strategy in the long-term management of pediatric respiratory infections.

### 4.3. Safety Considerations of Zinc Supplementation

Although zinc is generally well tolerated at recommended pediatric doses, chronic high-dose supplementation may pose risks. Excessive zinc intake can interfere with copper absorption, potentially leading to hypocupremia, anemia, and neutropenia [10,12]. Gastrointestinal side effects such as nausea, vomiting, abdominal pain, and metallic taste have also been reported in supplementation studies [22]. Most trials in pediatric respiratory infections employed short-to-moderate supplementation regimens and did not observe serious adverse events; however, long-term safety data remain limited, particularly for multicomponent formulations where cumulative micronutrient exposure is not always fully characterized [7,35].

### 4.4. Limitations and Future Research Directions

Despite the growing body of evidence supporting a role for zinc in pediatric respiratory infections, several important limitations must be considered. Substantial heterogeneity exists across studies in terms of zinc formulation, dosage, timing of administration, duration of supplementation, and bioavailability, making direct comparisons difficult and limiting the formulation of standardized clinical recommendations. Furthermore, many preventive trials were conducted in regions with a high prevalence of nutritional deficiency, a context in which the observed benefit may be amplified and therefore less generalizable to well-nourished pediatric populations [12]. In contrast, therapeutic studies conducted during acute infections frequently report inconsistent outcomes, partly due to small sample sizes, variability in disease severity at enrollment, and reliance on surrogate or intermediate endpoints rather than robust clinical outcomes [8]. Methodological constraints related to the literature search must also be acknowledged. Only English-language studies indexed in PubMed and the Cochrane Library were included, potentially leading to the omission of relevant data published in other languages or available in regional, non-indexed databases. In addition, the evidence base encompasses infants, preschool children, school-aged children, and adolescents, whose immune systems differ substantially in terms of maturation, functional capacity, and susceptibility to infection. These developmental differences may influence both disease course and response to zinc supplementation, thereby limiting direct comparability across age groups.

Another key limitation concerns the nature of the interventions investigated. A considerable proportion of available evidence derives from multicomponent nutraceutical formulations, in which zinc is combined with other bioactive substances such as vitamin D, β-glucans, or plant-derived compounds, making it difficult to isolate the specific contribution of zinc to observed clinical effects [9]. Although real-world and observational studies provide valuable insight into everyday clinical practice, they remain inherently susceptible to confounding, selection bias, recall bias, and variability in diagnostic definitions, and often lack microbiological confirmation of infections. Additional uncertainties relate to nutritional assessment and safety monitoring. Few studies include systematic evaluation of baseline dietary intake, adherence to supplementation, or concurrent use of other micronutrients. Long-term safety outcomes are also insufficiently characterized, particularly with respect to chronic or high-dose supplementation. Mechanistic understanding remains incomplete as well. Questions persist regarding zinc redistribution during acute inflammation, interactions with other micronutrients, and the reliability of commonly used biomarkers of zinc status in the presence of infection or systemic inflammation [11]. Moreover, dose–response relationships across different pediatric age groups and clinical phenotypes have not yet been clearly defined.

#### Future Research Directions

Future research should prioritize well-designed, adequately powered randomized controlled trials employing standardized dosing protocols, stratification according to baseline zinc concentration, and clinically meaningful endpoints. Comparative studies directly evaluating zinc monotherapy versus multicomponent formulations are needed to clarify causality and determine whether observed benefits are zinc-specific or mediated through synergistic interactions. Greater attention should also be devoted to age-specific immune responses, long-term safety monitoring, and the development of reliable biomarkers of zinc status during inflammatory states. Finally, population-level strategies such as food fortification and agricultural biofortification warrant further investigation with regard to their effectiveness, sustainability, and impact on infection burden [16]. Collectively, these efforts are essential to refine clinical indications, optimize supplementation strategies, and translate zinc research into rational, evidence-based practice in pediatric respiratory infections.

## 5. Conclusions

Zinc emerges as a biologically plausible and clinically relevant micronutrient in the context of pediatric respiratory infections. Across the available literature, preventive supplementation, particularly in deficiency-prone populations, is consistently associated with reduced incidence, recurrence, and overall morbidity of respiratory tract infections. These findings are reinforced by high-quality systematic reviews and Cochrane analyses, which indicate that zinc is most effective when used to restore adequate nutritional status and support immune competence over time, rather than as a stand-alone therapeutic intervention during acute disease. By contrast, the use of zinc as an adjunct treatment in acute bronchiolitis or pneumonia yields heterogeneous and largely inconclusive results, with limited impact on major clinical outcomes such as mortality, treatment failure, or length of hospitalization. These discrepancies likely reflect biological factors, including redistribution of zinc during systemic inflammation, as well as methodological heterogeneity across studies. Nevertheless, selected pediatric subgroups, particularly children with recurrent upper airway infections and tonsillitis, may derive benefit from zinc-containing multimodal nutraceuticals, especially when used within preventive or supportive strategies. Overall, the accumulated evidence supports a pragmatic interpretation: zinc should be considered a cornerstone of immune nutritional support in childhood, with a primary role in prevention and in reducing the burden of recurrent disease, while its therapeutic role during acute infections remains to be clearly defined. Future research should prioritize standardized dosing regimens, careful assessment of baseline zinc status, separation of zinc monotherapy from multicomponent formulations, and robust clinical endpoints. Integrating mechanistic biomarkers with high-quality randomized trials will be crucial to clarify which patients benefit most, at what dose, and in which clinical scenarios. Taken together, optimizing zinc status represents a safe, accessible, and potentially impactful strategy to complement existing approaches for the prevention and management of pediatric respiratory infections, particularly in populations at risk of deficiency.

## Data Availability

The original contributions presented in the study are included in the article, further inquiries can be directed to the corresponding author.
